# Radiation exposure in fluoroscopy guided spinal interventions: A prospective observational study of standard practice in a physiatry academic center^☆^

**DOI:** 10.1016/j.inpm.2023.100273

**Published:** 2023-08-09

**Authors:** Don Nguyen, Frédérique Piché, Christopher Mares, Isabelle Denis

**Affiliations:** a Physical Medicine and Rehabilitation, Faculty of Medicine, Université de Montréal, Québec, Canada; b Physical Medicine and Rehabilitation, Centre Hospitalier de l'Université de Montréal, Québec, Canada; c Radiation Safety Department, Centre Hospitalier de l'Université de Montréal, Québec, Canada

**Keywords:** Physiatry, Interventional, Pain, Physician, Radiation, Exposure, Fluoroscopy, Spine, Intervention, Injection, Finger, Hand, Eyes

## Abstract

**Context:**

Fluoroscopy is the recommended image guidance modality for most spinal pain interventions. However, it exposes interventional pain physicians to chronic ionizing radiation, with known risks to the eye, skin, and hand. The use of protective glasses and gloves is variable among pain physicians.

**Objectives:**

To document the total radiation exposure (mSv) by pain physicians to their eyes, hands and chest in an academic setting including various spinal interventions.

**Methods:**

Four pain physicians wore a finger, head/collar (equivalent to eye exposure) and chest dosimeter over and under their lead apron during a three-month period where they performed their usual fluoroscopy-guided interventions, including close supervision of trainees. We calculated an average exposure per intervention and extrapolated the recorded exposure to a maximum, worst-case scenario of a caseload of 13 procedures per day, 5 days a week and 52 weeks per year.

**Results:**

Four pain physicians of variable experience performed 15 different types of procedures on 607 patients throughout the study period. The yearly maximum exposure scenarios for each pain physician were all below the Canadian Nuclear Safety Commission thresholds for nuclear energy workers: for the hands (31.56 mSv, 25.67 mSv, 20.59 mSv, 21.51 mSv; threshold = 500 mSv), eyes (16.01 mSv, 18.64 mSv, 24.08 mSv, 18.68 mSv; threshold = 50 mSv) and chest over the lead apron (28.27 mSv, 46.91 mSv, 30.00 mSv, 40,03 mSv; whole body threshold = 50 mSv), with some doses even below general population thresholds. The exposure under the lead apron was 0 mSv for each pain physician.

**Conclusion:**

The standard practice of an interventional pain physician using fluoroscopy in this academic setting is below radiation exposure regulations, even in a theoretical, worst-case, maximum exposure scenario. Standard radiation protection practices such as the use of lead aprons and thyroid shields should still be used. However, this data is reassuring for pain physicians with a practice in fluoroscopy-guided interventions who wish to not use protective glasses or gloves.

## 1 Introduction

Fluoroscopy-guided interventions (FGI) are commonly performed by pain physicians who specialize in spinal interventions to treat radicular, facetogenic and sacroiliac pain [1]. They are usually performed by various medical specialists, including physiatrists. The range of procedures that can be performed under X-ray guidance includes, among others, transforaminal epidural steroid injections (TFESI), caudal epidural steroid injections (CESI), facet joint blocks (FJB), sacroiliac joint blocks (SIJB), medial branch blocks (MBB), lateral branch blocks (LBB) and radiofrequency ablations (RFA). According to the Spine Intervention Society (SIS), fluoroscopy is currently the recommended image guidance modality for most spinal interventions performed to treat pain, as its safety and validity are most documented in the medical literature [2]. Real-time fluoroscopy gives the ability to document contrast media flow and therefore ensures that medication injectate is delivered to the target space and excludes vascular or intrathecal uptakes [2]. It is also less affected by patients’ body habitus than ultrasound as a guidance method.

However, FGIs also expose the pain physician to chronic ionizing radiation. Studies have shown increased risk, albeit through a stochastic effect, to medical workers exposed to chronic ionizing radiation, for eyes (cataracts) [[3], [4], [5], [6]] and thyroid conditions (adenoma, thyroiditis, neoplasm) [7,8]. With skin, there is a known deterministic effect, with dermatitis occurring when absorbed radiation doses reach over 2 Gy [9]. Therefore, recommendations regarding yearly radiation dose limits are emitted by radiation protection governing commissions (Table 1, Table 2). Extremities and eyes dose limits are expressed within Equivalent Dose Limits, as they account for adjusted absorption doses to each organ, while whole body dose limits are expressed within Effective Dose Limits, representing the addition of equivalent doses to all organs [10]. In current practice, almost all physicians use a lead apron while performing FGI, but protective glasses, thyroid shield and lead gloves uses are more variable [4,7,8]. Studies have shown that protective glasses can reduce 70%–98% of the amount of perceived radiation from the fluoroscopic emitter, while the thyroid shield can reduce up to 70 times the exposure [3].

It is therefore important to document how much radiation is perceived at the eyes, hands and chest by pain physicians who specialize in FGIs, especially for those for who it is their main scope of practice. Other studies have shown safe exposure to the thyroid, eyes and hands for pain physicians performing specific techniques like TFESIs, MBBs and discographies under fluoroscopic guidance [[13], [14], [15]]. However, our literature review has not shown any publication studying a complete standard practice of a pain physician, which includes a vast range of procedures.

The goal of our study was to document the radiation perceived by interventional pain physicians in an academic setting, to the eyes and hands as the primary outcome, as their shielding is less commonly used, and to compare them to radiation dose limits emitted by the Canadian Nuclear Safety Commission for the general population and nuclear energy workers. Our secondary goal was to document the effectiveness of the lead apron in protecting the body. We expected that current radiation protection practice in pain medicine was within recommendations for nuclear energy workers but not necessarily for the general population.

## 2 Methodology

### 2.1 Setting and data sources

This prospective observational study was approved by the Research Ethics Board of the Centre Hospitalier de l’Université de Montréal (CHUM) (2023–10592, 22.103–YP). It was conducted at the physiatry fluoroscopy clinic of the CHUM, a tertiary academic hospital in Quebec, Canada. The clinic operates two similar fluoroscopy rooms, with two similar C-Arms machines (Phillips© BV Pulsera). All four physiatrists with a practice in FGIs at the CHUM were recruited through a written consent form. None were excluded. All were supervising a common fellow trainee in fluoroscopy, as well as regular physiatry residents through their clinics.

The pain physicians all wore a total of four dosimeters during each procedure for each patient (finger, eyes, over-apron chest, under-apron chest). They wore the finger dosimeter on their dominant hand, the under-apron chest dosimeter on their scrub pocket and the over-apron chest dosimeter on the apron external pocket. For three pain physicians (pain physician #2,#3,#4), the eye dosimeter was worn over the upper thyroid shield to substitute for the eyes, as our centre did not have eye dosimeters designed for glasses and the regular dosimeters were too heavy to be worn directly on the glasses. One pain physician (pain physician #1) wore the eye dosimeter in the fold of his scrub cap on the right temple.

The dosimeters were provided by the Radiation Safety Department of our center. The study lasted for three months, from October 1st, 2022, to December 31st, 2022. Each pain physician protected themselves as they would usually do. All four wore a lead apron and a thyroid shield. Two out of four wore protective glasses. None wore protective lead gloves.

Over the study period, the pain physicians performed their FGIs as usual, with no restriction to any technique they would usually perform. Every procedure done was included in the study. Cases with patients referred to the fluoroscopy clinic but ending up not having FGIs were excluded, as well as procedures done independently by a fellow trainee, without the attending pain physician present during the procedure. For each procedure, the radiology technician compiled the following data: identification of the pain physician who performed the procedure, date of the procedure, type of FGIs, number of vertebral levels or joints treated and if the pain physician performed the FGIs or if they were closely supervising the fellow trainee or a resident. Additional data provided by the fluoroscopy emitter were also noted such as the fluoroscopy time per procedure (in min:s) and the radiation emitted by the fluoroscopy emitter (in mGy). Patients' file numbers were kept for double verification of fluoroscopy data through the hospital database afterwards should it be needed.

All the FGIs were done with the use of a C-Arm machine with anterior-posterior (AP) and lateral X-ray image views. Lateral views were obtained with the fluoroscopy emitter working on the right side of the procedural table. The pain physicians were also standing on the right side during all of the FGIs. Each FGI was done according to the Spine Intervention Society (SIS) guidelines, including use of contrast media under live fluoroscopy and digital subtraction angiography in warranted cases. The procedural tables were equipped with lead table scatter shields covering both sides of the fluoroscopy emitter when used for the AP view.

To ensure reliable data a compliance registry was kept in each fluoroscopy suite and pain physicians had to checkmark the days when they performed FGIs and if they wore each of the four dosimeters that day. The dosimeters were kept in a separate room, in the pain physicians’ office, when not used. Pain physicians were instructed to only wear them during their FGIs.

At the end of the study period, the dosimeters were sent back to the Radiation Safety Department of our center for analysis and total radiation doses (in mSv) over the semester (three months) were provided for each pain physician for every dosimeter. We then extrapolated the data to a theoretical, maximal, "worst-case” scenario caseload of 13 patients per day (the average maximal caseload scheduled in our academic center per pain physician per day), 5 days a week (the opening days of our center), 52 weeks per year, to provide a maximal radiation exposure scenario, and compared them with yearly regulation limits.

### 2.2 Statistical analysis

Descriptive and numeral values were analyzed with Microsoft Excel. All total, averages and percentage values were calculated directly throughout the spreadsheets. Continuous variables were presented as means with ranges. To get an average radiation exposure per dosimeter per procedure, we first took the total radiation value from the whole semester provided per dosimeter. We then divided the total by the number of procedures in which each dosimeter was worn. With an average radiation exposure per dosimeter per procedure, we could then extrapolate the values to a yearly maximal, worst-case scenario of FGIs and compare them with the Year Equivalent Dose Limits listed in Table 1, for the eye and finger dosimeters, as well as with the Effective Dose Limits listed in Table 2, for the over-apron chest dosimeters and the under-apron chest dosimeters.

## 3 Results

The study group consisted of four pain physicians specializing in FGIs, two women and two men, with experience ranging from one year to twenty-five years. During the study period, they all regularly worked at the fluoroscopy clinic (range of 13–24 days). 607 FGIs were performed during the study period, with an average of 8.32 procedures (range 7.22–9.78) performed per pain physician per day, over a usual caseload of 13 patients scheduled per day.

The pain physicians all performed a variety of FGIs: TFESI, CESI, FJB, SIJ, MBB, LBB and RFA, with some exceptions (Table 3). The most common procedures were cervical/thoracic/lumbar FJBs and SIJBs (384/607), followed by cervical and lumbar TFESIs and CESIs (122/607), cervical and lumbar MBBs and LBBs (53/607), and all RFAs (35/607). A total of 562 procedures were recorded with the finger dosimeters and 530 procedures were recorded with the eyes, over-apron chest and under-apron chest dosimeters.

**Table 1:** Equivalent dose limits^a^.

ORGAN OR TISSUE	PERSON	PERIOD	EQUIVALENT DOSE (MSV)
**Lens of an eye**	Nuclear energy worker	One-year dosimetry period	50
	Any other person	One calendar year	15
**Skin**	Nuclear energy worker	One-year dosimetry period	500
	Any other person	One calendar year	50
**Hands and feet**	Nuclear energy worker	One-year dosimetry period	500
	Any other person	One calendar year	50

a Adapted from the Canadian Nuclear Safety Commission [11].

**Table 2:** Effective dose limits^a^.

PERSON	PERIOD	EQUIVALENT DOSE (MSV)
**Nuclear energy worker, including a female nuclear energy worker who is breastfeeding and a female nuclear energy worker who is pregnant but who has not yet informed the licensee in writing that she is pregnant**	One-year dosimetry period	50
	Five-year dosimetry period	100
**Pregnant nuclear energy worker who has informed the licensee in writing that she is pregnant**	Balance of the pregnancy starting from the date on which the licensee has been informed of the pregnancy	4
**Person who is not a nuclear energy worker**	One calendar year	1

a Adapted from the Canadian Nuclear Safety Commission [12].

**Table 3:** Procedures data.

		Pain physician			
		#1	#2	#3	#4
Fluoroscopy time per FGIs (s)		73.16	62.23	78.52	103.87
				

Number of FGIs per type of procedure	Cervical FJB	39	27	15	27
	Thoracic/thoracolumbar FJB	5	7	5	7
	Lumbar FJB	80	73	26	49
	SIJB	10	3	7	4

	CESI	5	10	6	8
	Cervical TFESI	0	1	0	1
	Thoracic TFESI	0	2	0	0
	Lumbar TFESI	30	31	16	12

	Cervical MBB	9	7	0	6
	Thoracic MBB	0	0	0	0
	Lumbar MBB	8	5	9	9
	LBB	0	0	0	0

	Cervical RFA	1	2	5	1
	Thoracic RFA	1	0	0	0
	Lumbar RFA	10	5	6	3
	SIJ RFA	1	0	0	0
	Others^a^	6	3	1	3

Total number of FGIs in study period		205	176	96	130
FGI operators	Number of FGIs performed by pain physicians	173	155	63	82
	Number of supervised FGIs performed by trainees	32	21	33	48
	Percentage of FGIs performed by pain physicians	84.4%	88.1%	65.6%	63.1%

a Including: knee RFA, sacrococcygeal block, trigger points, multiple procedures combined.

The average fluoroscopy time was 77,41 s per pain physician (average range of 62.23 s–103.87 s).

The pain physicians performed 77.9% of the FGIs themselves and supervised the rest (range of 63.1%–84.4% per pain physician).

With a theoretical, maximal worst-case scenario extrapolation of 13 FGIs a day, five days a week, for 52 weeks a year, the pain physicians would have performed 3380 FGIs per year. Over the study period, the pain physicians had respectively a total of 1.83 mSv, 1.2 mSv, 0.53 mSv, 0.53 mSv recorded for the finger dosimeter and respectively a total of 0.81 mSv, 0.91 mSv, 0.57 mSv and 0.63 mSv for the eye-equivalent thyroid shield dosimeter. When divided by the number of FGIs performed with the dosimeters and extrapolated for 3380 procedures, the pain physicians had respectively a projected maximal yearly exposure of 31.56 mSv, 25.67 mSv, 20.59 mSv, 21.51 mSv for the finger dosimeter and 16.01 mSv, 18.64 mSv, 24.08 mSv, 18.68 mSv for the eye dosimeter (Table 4).

**Table 4:** Dosimetry data.

		Pain physician			
	Dosimeter	#1	#2	#3	#4
Total exposure recorded during study period (mSv)	Finger	1.83	1.2	0.53	0.77
	Eye	0.81	0.91	0.57	0.63
	Over-apron chest	1.43	2.29	0.71	1.35
	Under-apron chest	0	0	No data^a^	0

Maximum yearly exposure scenario (mSv)	Finger	31.56	25.67	20.59	21.51
	Eye	16.01	18.64	24.08	18.68
	Over-apron chest	28.27	46.91	30.00	40.03
	Under-apron chest	0.00	0.00	No data^a^	0.00

Total FGIs recorded with dosimeters	Finger	196	158	87	121
	Eyes, over-apron chest, under-apron chest	171	165	80	114

a Dosimeter was lost.

The projected eye exposure was lower for pain physician #1 (16.01 mSv), who wore the eye dosimeter in the fold of their scrub cap than for the other three pain physicians (18.64, 24.08, 18.68), who wore it on their thyroid shield (Table 4).

Over the study period, the pain physicians also had respectively a total of 1.43 mSv, 2.29 mSv, 0.71 mSv and 1.35 mSv recorded on the over-apron chest dosimeter, leading to a maximal scenario of 28.27 mSv, 46.91 mSv, 30.00 mSv, 40,03 mSv. The exposure of the under-apron chest dosimeter was 0 mSv for each pain physician, although one under-apron chest dosimeter was lost when sent back for analysis and could not provide results (Table 4).

## 4 Discussion

This is the first study to our knowledge to measure the total radiation perceived by interventional pain physicians with a practice in FGIs through a full regular practice, including all spine levels of FJBs, TFESIs, MBBs, RFAs, SIJBs and LBBs. We used a worst-case scenario projection model over 52 weeks of full-time work per year and even in that scenario, we showed that the radiation exposures to pain physicians were well within the threshold limits of nuclear energy workers provided by the Canadian Nuclear Safety Commission for the eyes and the hands. Some of our values were even in the normal range for the general population. This is consistent with previous studies which included specific interventions (epidurals, discography) [[13], [14], [15], [16]] and confirm that the scattered radiation perceived by pain physicians during their practice in FGIs is therefore safe according to nuclear safety regulations. The FGIs range in this study was diverse, included close supervision of fellow trainees and residents, and the three-month study period was long enough to have a representative caseload of a pain physician with a practice in FGIs in an academic center.

According to our results, the use of lead gloves to protect the hands might not be strictly warranted. One study had shown that their protective effect was marginally efficient [17]. The gloves could still be used to maximize protection, according to pain physicians' preferences. Some could be bothered by the diminishing dexterity and discomfort with their use. The “As Low As Reasonably Achievable” (ALARA) radiation protection concept still needs to be applied and we suggest that when the needle is being repositioned, to hold the hand back as much as possible during the X-ray images, as suggested by other studies [8,[18], [19], [20]].

We did not have glass dosimeters to directly measure the eye exposure in our center and had to use the extrapolation of the upper neck radiation from the thyroid shield dosimeter or fold of the scrub cap dosimeter. The perceived radiation from the neck and eyes differs between the pain physician's practice, depending on their height, posture, and positioning, as shown in our study by the pain physician who wore the dosimeter on the fold of his scrub cap. This dosimeter location is likely the most representative of actual eye lens perceived radiation and is further from the emitter, which likely accounts for the lower perceived dose compared to the pain physicians who wore it on the thyroid shield. While that perceived dose was lower, the difference was however not large, and all yearly maximal exposure scenarios from all four pain physicians were within the same Yearly Equivalent Dose limit thresholds. This correlates with what was found in a recent study, in which the exposure was also greater when measured on the thyroid shield than on the scrub cap [16]. Using the “worst-case scenario” perspective, the actual eye exposure could therefore be even lower in our study for pain physicians #2, #3 and #4. With this data, protective glasses may therefore not be strictly warranted.

However, it would still be reasonable to use protective glasses regularly. The maximal exposure scenario in our study still shows above recommended exposure limits for the lens of an eye for the general population, even if they would be within limits for nuclear energy workers. Some studies have shown an increased relative risk of cataracts among medical workers exposed to chronic ionizing radiation through a stochastic effect [5,6]. A recent study also showed that protective eyewear is efficient to reduce eye radiation exposure [21]. To further limit the eye exposure, we would also recommend standing away from the beam during use of fluoroscopy, concordant with the ALARA principle.

The lead aprons seemed to be effective in blocking scattered radiation to the chest as in most studies [13,14,18]. Some studies study showed low scattered radiation below the apron but this could have been due to inadequate apron wearing or sizing [18].

Our center was also equipped with lead table scatter shields, which may have reduced the overall radiation exposures, by blocking parts of the leakage X-rays from the primary beam of the fluoroscopy emitter. However, they were only covering the fluoroscopy emitter when the C-Arm was used for AP views, as the fluoroscopy emitter was exposed during lateral views due to the inclining of the emitter. We are therefore unable to quantify the magnitude of its effect. A recent study also showed that covering the fluoroscopy emitter altogether with a lead apron can reduce radiation exposure, which was not done in this study [22].

### 4.1 Limitations

One of the main limitations of our study was that we did not measure the distance where the pain physicians were standing from the fluoroscopy emitter, nor from the beam's entry point in the patient which differs between AP and lateral views. It is known in the literature that distance is one of the factors influencing perceived scattered radiation [18]. We are aware that the distance from the beam could have also varied a lot depending on the various techniques included in our study, but it was representative of regular practice. As the pain physicians in our study were standing on the same side as the fluoroscopy emitter during the lateral image acquisitions, they were also probably exposed to significantly more scatter radiation than if they had been on the image receptor side. This is a practice that may already be done in other practice settings and could lessen the perceived scattered radiation. Because of the nature of our academic setting, the pain physicians also did not directly perform all of the FGIs, although they were close to trainees in all of them. This could have further affected the distances and exposure, as pain physicians #1 and #2, who performed a larger percentage of FGIs themselves (84.4% and 88.1%), had more finger exposure than pain physicians #3 and #4. However, those differences were not large and there were no difference tendencies regarding the other dosimeters. Trainees are a mainstay of academic practice, and this was representative of such. Further studies could be done to better quantify the impact of trainee supervision, as distance, but also other factors like fluoroscopic time, number of images taken and complexity of techniques, can affect the exposure.

We did not measure the Body Mass Index of patients either, even if it is known that they can affect the radiation emitted by the fluoroscopy emitter and the fluoroscopic time [23]. As the scope of this study was observative and not comparative, we decided that controlling this variable was not needed. Another limitation of the study is that in our academic center, the number of scheduled FGIs patients is usually thirteen per day on average. In other non-academic settings, this caseload can be much higher, leading to a potential underestimation of the exposure of a pain physician performing FGIs.

We did not assess the standard radiation protection precautions taken by the pain physicians in this study, although there are regular radiation protection courses every three years in our academic center. It is well known that the use of pulsed fluoroscopy versus continuous technique and minimizing the amount of image taken lessen the scattered radiation. Standing back between the images is also recommended. These measures might and probably have been individually taken and contributed to the within-limit values. Because this is an observational, non-comparative and non-blinded study, pain physicians were aware of the study settings. Therefore, they may have been biased and been more careful with their radiation protection practices during the study period than during their regular practice.

The average fluoroscopy times were longer in this study than others that we found in the literature [20,24,25]. Many factors could have influenced that. First, this study was conducted in a tertiary academic hospital with trainees. This setting leads to more images taken for teaching purposes and trainee supervision which takes more fluoroscopy time. The range of procedures was wider, with more complex patients, and included longer procedures with more vertebral levels treated. FJBs, especially lumbar, were the most performed procedure, requiring up to four to six injections, leading to more fluoroscopy time than a single or double TFESI. Also, lumbar RFAs are longer procedures and require more fluoroscopy images for needle placement.

## 5 Conclusion

In conclusion, the total scattered radiation perceived by the eyes and hands are well below the recommendations of the Canadian Nuclear Safety Commission for nuclear energy workers in a global practice of interventional pain physicians with a practice in FGIs, even in a maximal, theoretical, worst-case scenario caseload. Lead body aprons are also effective in blocking scattered radiation. Principles of radiation protection are warranted, as well as the ALARA principles. However, this data is reassuring for pain physicians with a practice in FGIs not using protective glasses or gloves. Further studies could be done with specific eye dosimeters with the same settings, as well as non-academic settings with an even greater FGIs procedures caseload.

The authors declare that they have no known competing ﬁnancial interests or personal relationships that could have appeared to inﬂuence the work reported in this paper.

## Disclosure

The research team has no conflict of interest to declare. No financial support was provided for this research project.

## Declaration of competing interest

The authors declare that they have no known competing financial interests or personal relationships that could have appeared to influence the work reported in this paper.
